# Getting Serious About the Prevention of Chronic Diseases

**Published:** 2011-06-15

**Authors:** Nicholas Freudenberg, Kenneth Olden

**Affiliations:** City University of New York School of Public Health at Hunter College; City University of New York School of Public Health at Hunter College, New York, New York

Passage of the Patient Protection and Affordable Care Act of 2010 marks an important step toward making health care available to all Americans. However, implementation of the legislation over the next decade faces organizational, political, and economic challenges (1). One of the surest ways to maximize the chances for health care reform to achieve its aims is to reduce the burden of chronic disease on the nation’s health care system.

Chronic diseases such as heart disease, cancer, hypertension, stroke, and diabetes now account for 80% of deaths in the United States and 75% of health care costs. In 2005, 44% of all Americans had at least 1 chronic condition and 13% had 3 or more. By 2020, an estimated 157 million US residents will have 1 chronic condition or more (2). Although an aging population has contributed to the increase in chronic conditions, children and young adults face a growing prevalence of obesity, diabetes, and asthma. Between 1996 and 2005, the number of people aged 25 to 44 years with more than 1 chronic disease doubled (2).

Health care reform legislation promises better access to screening and early intervention for chronic conditions for vulnerable populations. Similarly, advances in understanding the role of the human genome in the expression of chronic conditions offers hope for new treatments (3). Unfortunately, evidence suggests that innovations in genomic medicine are unlikely to reduce the prevalence or costs of chronic conditions in the coming decade (4).

To lower the incidence of chronic diseases and thus the costs they impose on our society and health care system will require addressing the deeper causes of the increase in recent decades. Much evidence suggests concrete action that could help to prevent further increases in chronic diseases. We suggest 4 broad strategies.

First, the United States needs to bring its environmental and consumer protection regulations into the 21st century. Air pollution, especially in urban and low-income areas, contributes to illnesses and deaths from cancer and heart and respiratory diseases. In 2002, at least 146 million people in the United States lived in areas that did not meet at least 1 US Environmental Protection Agency air pollution standard (5). Tobacco and alcohol use and consumption of foods high in fat, sugar, salt, and calories contribute to a substantial proportion of chronic disease deaths, yet most of the nation’s regulatory approaches to these products were developed in the first half of the 20th century. New forms of marketing, product design, and retail distribution make these old approaches insufficient to protect the population against the aggressive promotion of these unhealthful products. In the last 3 decades, the tobacco, alcohol, and food industries have substantially increased their efforts to oppose public health protection against these products and to persuade consumers to use their products (6). Developing stronger national, state, and local protections and finding new ways to prevent these industries from externalizing their costs onto taxpayers can contribute to reducing the behaviors that put people at risk for chronic diseases (7).

Second, the nation needs to maintain and strengthen federal, state, and local public health infrastructure. In 2004, Frieden charged local public health officials in the United States with being “asleep at the switch” in their response to the growing threats from chronic disease (8). He urged stronger surveillance programs, environmental interventions, new regulation, and more funding. In the past 2 years, however, as a result of the economic crisis, many state and local health departments have cut funding for services, including chronic disease control (9). This action jeopardizes prevention of chronic diseases by increasing the flow of people with chronic illnesses into the health care system, making it more difficult for health reform to achieve its objectives.

Third, the country needs to offer new incentives to create a built environment that promotes health. Increased physical activity protects against several chronic conditions, yet urban, suburban, and rural environments often make it difficult to walk, bicycle, or use other forms of active transport. Government can help people make more healthful choices the default choice by modifying zoning rules; developing transportation systems that encourage active transport; and designing schools, workplaces, and communities that promote physical activity and discourage being sedentary.

Finally, the nation’s health care system needs to modify its practices to make prevention of chronic diseases a priority. This modification could be achieved by extending the reach of evidence-based intervention programs; strengthening community health centers; increasing reimbursement for services such as tobacco use cessation, nutrition, and alcohol counseling; and providing health professionals with additional prevention skills (10-12).

These 4 strategies offer several advantages. They rely on off-the-shelf science, reducing the necessity of additional years of research before implementation. Each contributes to improvements in various chronic conditions. The proposed measures can contribute to reductions in cancer, diabetes, hypertension, and heart disease (Table), each projected to increase in prevalence by more than 40% in the next 2 decades (10). In addition, these strategies reduce population incidence of chronic conditions and help shrink disparities because the targeted conditions mostly affect low-income, black, and Latino populations.

Updating environmental and consumer protection, strengthening the public health infrastructure, improving the built environment that influences health, and making prevention a priority for our health care system have the potential to win broad voter and policy maker support. Although any policy reforms that threaten the status quo will be opposed by some special interests, these recommendations will benefit most people in the United States, save taxpayer dollars, and help the nation to achieve its health goals. Implementing the 4 simultaneously will help to achieve synergies that can accelerate and magnify their effect. By providing the leadership needed to realize these changes, health professionals can increase the likelihood that health care reform will succeed and that the nation’s health will improve.

## Figures and Tables

**Table.** Public Health Strategies to Prevent Cardiovascular and Respiratory Diseases, Some Cancers, Diabetes, and Other Chronic Conditions

**Strategy**	Specific Objectives
Strengthen and update consumer and environmental protection	Reduce fat, sugar, and salt in food; reduce promotion of unhealthful food; reduce air pollution; reduce tobacco use; restrict expanded promotion of alcohol and tobacco to youth.
Support public health infrastructure	Strengthen screening for risk conditions; fund local and state health departments for sustainable, community-based comprehensive campaigns against chronic diseases; establish and strengthen chronic disease surveillance programs to inform prevention; prevent budget cuts that undermine infrastructure.
Improve built environment	Encourage walking, biking, and other forms of active transportation; develop design guidelines that support active living in schools, communities, and workplaces; use zoning to improve food and physical activity environments; encourage mass transit.
Make prevention a health care priority	Provide financial and organizational incentives for primary prevention by using evidence-based interventions; improve training of primary care providers; develop stronger links between public health and health care systems; strengthen community health centers.

## References

[1] Skocpol T (2010). The political challenges that may undermine health reform. Health Aff (Millwood).

[2] (2009). Tackling the burden of chronic diseases in the USA. Lancet.

[3] Scheuner MT, Sieverding P, Shekelle PG (2008). Delivery of genomic medicine for common chronic adult diseases: a systematic review. JAMA.

[4] Russell LB (2009). Preventing chronic disease: an important investment, but don't count on cost savings. Health Aff (Millwood).

[5] Kim JJ, American Academy of Pediatrics Committee on Environmental Health (2004). Ambient air pollution: health hazards to children. Pediatrics.

[6] Vladeck D, Weber G (2004). Commercial speech and the public's health: regulating advertisements of tobacco, alcohol, high fat foods and other potentially hazardous products. J Law Med Ethics.

[7] Olden K, Ramos RM, Freudenberg N (2009). To reduce urban disparities in health, strengthen and enforce equitably environmental and consumer laws. J Urban Health.

[8] Frieden TR (2004). Asleep at the switch: local public health and chronic disease. Am J Public Health.

[9] Levi J, St Laurent, Segal LM, Vinter S Shortchanging America's health: a state-by-state look at how public health dollars are spent and key state health facts.

[10] Bodenheimer T, Chen E, Bennett HD (2009). Confronting the growing burden of chronic disease: can the U.S. health care workforce do the job?. Health Aff (Millwood).

[11] EPC Team, Whitlock EP, O'Conner EA, Williams SB, Beil TL, Lutz KW Effectiveness of primary care interventions for weight management in children and adolescents: an updated, targeted systematic review for the USPSTF.

[12] Bertakis KD, Azari R (2007). Determinants of physician discussion regarding tobacco and alcohol abuse. J Health Commun.

